# Cancer therapy – molecular targets in tumour–host interactions

**DOI:** 10.1038/sj.bjc.6603045

**Published:** 2006-04-04

**Authors:** P Kurschat, C Mauch

**Affiliations:** 1 University of Cologne, Cologne, Germany

During the past few decades, cancer therapy was based on the classical options of surgery, radiation and chemotherapy. And although there are examples where these techniques are applied with great success, there is still a lack of good treatment options for the most common types of cancer, especially in advanced cases with distant organ metastasis.

During the last 20 years, basic research has considerably expanded our understanding of tumour biology. It has become obvious that cancer development and progression consists of several consecutive steps, and many molecular key events have been identified. One of the most important changes in our view of cancer in general was that we noticed the importance of tumour–host interactions. There is a constant cross-talk between tumour cells on one side and the surrounding stroma and immune cells on the other side. This is most obvious for tumour angiogenesis, but there are several other examples like the contribution of fibroblasts to the proteolytic capacity of the tumour or the various immune escape mechanisms used by the neoplastic cells to circumvent attack by the immune system.

Currently, several new approaches based on these discoveries are trying to make the step from laboratory to clinic. Many promising results were obtained from *in vitro* and animal experiments, indicating that specific disturbances in tumour–host interactions might slow down the progression of malignant disease. Therefore, it is challenging for everyone working in the field of oncology to keep up with the newest developments of anticancer strategies.

‘Cancer Therapy-Molecular Targets in Tumour-Host Interactions’ provides in 16 chapters examples of molecular key events and mechanisms during tumour progression, which might be targeted to interfere with malignant progression. Special emphasis is put on antiangiogenesis to prevent the formation of new blood vessels, leading to reduced tumour growth due to a lack of oxygen and nutrients. Most articles cite the literature until 2004, which is sufficient to cover newer developments.

Our main criticism concerning this book is that the reader does not really get an overview of the field. The preface by Georg F Weber is written well and leads into the field, but it is too short to really connect the following parts. The single chapters, all written by different authors, focus on very specialised topics, but largely fail to cover the broader field.

There is no common format, and some chapters resemble more lab protocols. The length of the different articles varies considerably between 10 and 50 pages, and at least to us it is not obvious on which criteria the different aspects of the field were weighted. One of the major shortcomings is the far too low number of figures. These could have been used to facilitate reading and understanding of the topics, especially for readers not working in the particular fields.

In summary, this book contains some interesting articles addressing specialised fields of research, and therefore it might be useful to readers searching for distinct topics. But we cannot recommend it for readers who want to get an overview over the field. The approach is not systematic enough and the single chapters focus too much on certain aspects, not covering the whole field in review style. Therefore, this book appears to be suitable only to a specialised audience.

